# A Diamond Temperature Sensor Based on the Energy Level Shift of Nitrogen-Vacancy Color Centers

**DOI:** 10.3390/nano9111576

**Published:** 2019-11-07

**Authors:** Mingyang Yang, Qilong Yuan, Jingyao Gao, Shengcheng Shu, Feiyue Chen, Huifang Sun, Kazuhito Nishimura, Shaolong Wang, Jian Yi, Cheng-Te Lin, Nan Jiang

**Affiliations:** 1 Key Laboratory of Marine Materials and Related Technologies, Zhejiang Key Laboratory of Marine Materials and Protective Technologies, Ningbo Institute of Materials Technology and Engineering (NIMTE), Chinese Academy of Sciences, Ningbo 315201, China; yangmingyang@nimte.ac.cn (M.Y.); gaojingyao@nimte.ac.cn (J.G.); shushengcheng@nimte.ac.cn (S.S.); chenfeiyue@nimte.ac.cn (F.C.); sunhuifang@nimte.ac.cn (H.S.); wangshaolong@nimte.ac.cn (S.W.); yijian@nimte.ac.cn (J.Y.); 2 Center of Materials Science and Optoelectronics Engineering, University of Chinese Academy of Sciences, Beijing 100049, China; 3 College of Science, Henan University of Technology, Zhengzhou 10463, China; 4 Faculty of Materials Science and Engineering, Kunming University of Science and Technology, Kunming 650093, China; 5 Advanced Nano-processing Engineering Lab, Mechanical Engineering, Kogakuin University, Tokyo 192-0015, Japan; nishimura@cc.kogakuin.ac.jp

**Keywords:** diamond temperature sensor, nitrogen-vacancy (NV) color center, temperature dependence, energy level shifts, zero-phonon line (ZPL), modified Varshni model

## Abstract

The nitrogen-vacancy (NV) color center in chemical vapor deposition (CVD) diamond has been widely investigated in quantum information and quantum biosensors due to its excellent photon emission stability and long spin coherence time. However, the temperature dependence of the energy level of NV color centers in diamond is different from other semiconductors with the same diamond cubic structure for the high Debye temperature and very small thermal expansion coefficient of diamond. In this work, a diamond sensor for temperature measurement with high precision was fabricated based on the investigation of the energy level shifts of NV centers by Raman and photoluminescence (PL) spectra. The results show that the intensity and linewidth of the zero-phonon line of NV centers highly depend on the environmental temperature, and the energy level shifts of NV centers in diamond follow the modified Varshni model very well, a model which is better than the traditional version. Accordingly, the NV color center shows the ability in temperature measurement with a high accuracy of up to 98%. The high dependence of NV centers on environmental temperature shows the possibility of temperature monitoring of NV center-based quantum sensors in biosystems.

## 1. Introduction

The recent fast development of quantum information science has pushed the wide investigation of nitrogen-vacancy (NV) color centers in diamond both experimentally and theoretically [1,2]. Due to their excellent photon emission stability and long spin coherence time [3,4], NV centers, especially NV^−^ centers, have attracted extensive attention in the area of quantum computation [5,6,7], quantum sensors [8,9,10], quantum imaging [11,12], and so on [13,14,15]. In diamond, the NV center is a point defect with a C_3V_ symmetry structure which is made up of a substitutional nitrogen atom and an adjacent vacancy center in the crystal lattice of diamond [16]. According to the charge states of NV centers, NV color centers have two types: NV^0^, which has electric neutrality, and NV^−^, which contains a negative electron. NV^0^ and NV^−^ have energy levels of about 2.156 and 1.945 eV below the conduction band of diamond and zero-phonon lines (ZPL) at 575 and 637 nm on the fluorescence emission spectra, respectively [17,18,19].

Nitrogen is the main impurity in diamond, and in many cases, nitrogen atoms exist in diamond as pairs or clusters by replacing carbon atoms as substitutional atoms; instead of forming NV centers, for example, the nitrogen atoms in type Ia diamond are usually in pairs [20]. In order to create NV centers in diamond, some methods have been developed. For example, nitrogen atoms can be implanted into ultrapure diamond following thermal annealing to create single or discrete NV defect centers in diamond [21], and nitrogen-containing diamond can also be irradiated by an electron dose followed by thermal annealing to convert NV^0^ into NV^−^ centers [22]. In addition, NV centers can also be created in chemical vapor deposition (CVD) diamond by introducing N_2_ gas during the synthesis process. Compared to other methods, the concentration of NV centers is very high, but NV centers can be uniformly distributed in CVD diamond from a macro perspective. As a result, CVD diamond with NV centers has found application within quantum biosensors for molecular tracking [23] and temperature monitoring [24].

Temperature sensors based on the fluorescence emission of NV centers in diamond have been widely researched in biosystems, not only due to the good biocompatibility of diamond but also the high sensitivity of NV centers to environmental temperature [25]. Most of these sensors are based on the electron spin resonances of NV^−^ centers in the presence of a magnetic field [9,26,27] and have a high requirement for experimental facilities for testing the optically detected magnetic resonances (ODMR) of NV^−^ centers, or the energy level shifts of NV centers under different temperatures [28]. According to traditional theory, the temperature-induced thermal expansion effect and the phonon-electron (acoustic or optical) interaction effect are the main factors causing the energy gap shift of conventional semiconductors like Si and Ge [29,30,31,32]. However, the temperature dependence of NV centers’ energy level in diamond is different from traditional semiconductors due to the very high Debye temperature (*θ_D_*: 2200 K) [33], large optical phonon frequency (165.2 meV) [34], and very small thermal expansion coefficient (1.6 × 10^−6^ K^−1^) [35]. While some works have presented investigations and applications of diamond temperature sensors based on NV centers [26,27], the dependence of the fluorescence emission properties of NV centers to temperature has not been clearly revealed and needs detailed investigation.

In this work, a diamond sensor for temperature measurement is proposed based on the energy level shifts of NV centers to temperature in diamond samples. The diamond samples with NV centers were epitaxially grown by microwave plasma chemical vapor deposition (MPCVD). The fluorescence emission spectra of NV centers were measured by Raman and photoluminescence (PL) spectroscopy in the temperature range of 80 K to 300 K. According to the results, the relation of the energy level of the NV centers to temperature follows the modified Varshni model. The diamond sensor for temperature measurement shows high accuracy of up to about 98% and 97% for NV^0^ and NV^−^ centers, respectively. The high temperature dependence and the highly accurate temperature measurement of NV centers reveal potential applications such as quantum biosensors in temperature detection and monitoring.

## 2. Experiments and Methods

Single-crystalline diamond samples were synthesized epitaxially on high pressure high temperature (HPHT) Ib-type (100) diamond substrate (Shenzhen Tiantian Xiangshang Diamond Co. Ltd. Shenzhen, China) via the MPCVD method. The chamber pressure for diamond growth was set at around 16 KPa and the temperature of the HPHT diamond substrate was maintained at around 930 °C. CH_4_ (4 sccm), which was used as the carbon source, was pumped into the CVD system for the growth of diamond together with H_2_ (400 sccm) as the carrier gas. For the formation of a nitrogen-vacancy center in epitaxial CVD diamond, N_2_ gas with different concentrations of 0 sccm (Sample A), 0.05 sccm (Sample B), and 10 sccm (Sample C) were introduced into the chamber during the synthesis process.

The crystallinity of the HPHT and CVD diamond samples were characterized by a confocal micro-Raman system with an excitation wavelength of 532 nm generated by an He-Ne laser (Renishaw, Renishawplc, Wotton-under-Edge, UK). The power of the laser (532 nm) used for Raman characterization was about 0.6 mW. The laser was focused on the diamond surface using a diameter of spot size of about 1.3 μm. The fluorescence of the NV centers was excited when exposed to the incident excitation laser. Meanwhile, fluorescence light was collected by the charge coupled device (CCD) to the spectrometer through the same microscope objective. Such equipment was also used to characterize the photoluminescence (PL) spectra of NV centers in diamond. The temperatures for the experimental measurements were controlled by a T95-LinkPad System Controller system (Linkam Scientific Instruments Ltd, Tadworth, UK) through liquid nitrogen. The measurement temperature applied to the sample was set between 80 to 300 K with the temperature fluctuation being smaller than 0.1 K.

## 3. Results and Discussion

### 3.1. Raman and PL Spectroscopy of Different Diamond Samples

**Figure 1:**
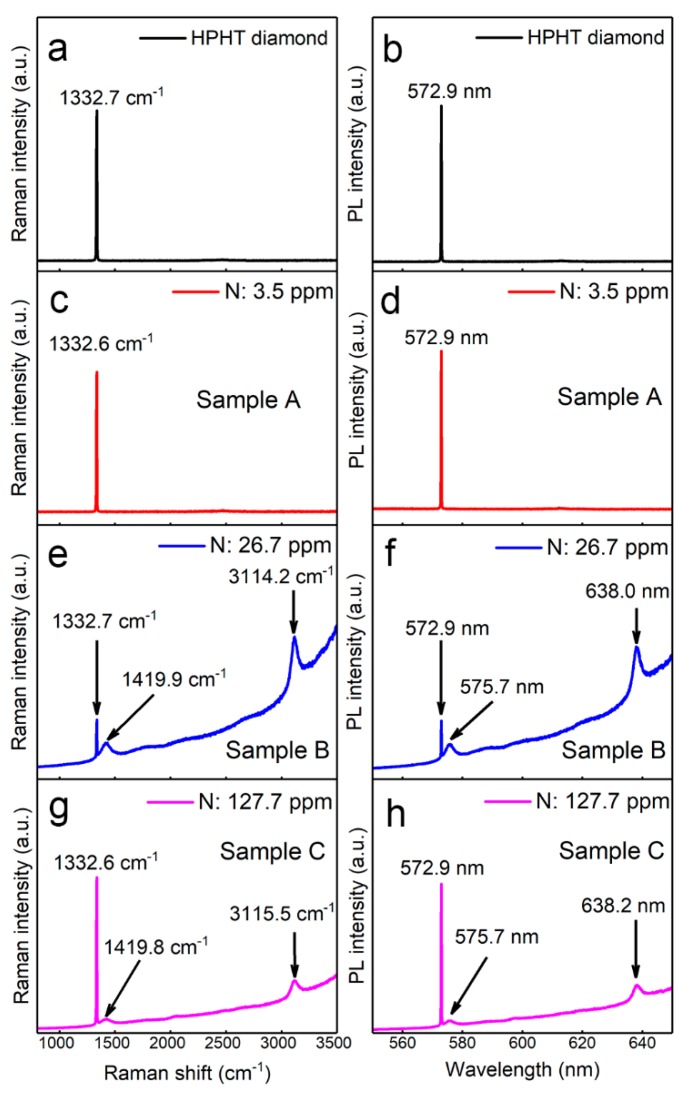
Raman and photoluminescence (PL) spectra of diamond samples in room temperature: (**a**,**b**) high pressure high temperature (HPHT) diamond and epitaxial chemical vapor deposition (CVD) diamond with nitrogen concentrations of (**c**,**d**) 3.5 ppm, (**e**,**f**) 26.7 ppm and (**g**,**h**) 127.7 ppm.

The diamond samples (Samples A, B, and C) with different nitrogen concentrations (Figure S1 and Table S1) before and after CVD epitaxial growth were characterized by Raman spectroscopy and PL spectroscopy, as shown in Figure 1. The first-order Raman scattering frequency of the HPHT diamond is located at around 1332.7 cm^−1^ in the Raman spectra and a peak located at 572.9 nm can also be observed in the PL spectra, as shown in Figure 1a,b, respectively. The Raman scattering in diamond is caused by the vibration of the two-interpenetrating face-centered cubic (fcc) lattices that comprise the diamond lattice, with one shifting one quarter along the diagonal of the other, which absorbs an energy of about 165 meV [36]. As a result, when excited by a 532 nm laser, a phonon in the ground state will absorb an incident photon (≈2.33 eV) and transit to the excitation state. When the phonon returns to the ground state, a photon with an energy of 2.165 eV will be released due to the lattice vibrational absorption of 165 meV. Hence, the released photon will lead to a diamond peak at 572.9 nm in the PL spectra and a corresponding 1332 cm^−1^ peak in the Raman spectra, respectively [37].

For the epitaxial CVD diamond (Sample A), without the introduction of N_2_ gas during growth, only the peak at 1332.6 cm^−1^ and the peak at 572.9 nm can be observed in the Raman and PL spectra, respectively, as shown in Figure 1c,d. When N_2_ gas was introduced into the CVD system during diamond growth (Samples B and C), as shown in Figure 1e–h, besides the diamond peak at 1332.7 cm^−1^, another two peaks can be also observed at around 1420 and 3115 cm^−1^ in the Raman spectra. The PL spectra show nearly the same profile to the Raman curve, and contain two peaks located at around 575.5 and 638.2 nm, respectively. The position of the peaks in the PL and Raman spectra follows

(1) $$E=\;\frac{1.24}{\lambda }\;,\;\Delta E=\;\frac{1.24}{\Delta \lambda }$$

(2) $$\Delta E_{\;}=\;E_{0}\;-\;E$$

(3) $$S_{R}=\;\frac{10^{4}}{\Delta \lambda \;}$$

where *λ* (μm) is wavelength of the incident or emission photon, *E* (eV) is the corresponding energy of the photons, *S_R_* (cm^−1^) is the Raman shift, and *E*_0_ (eV) is about 2.33 eV, corresponding to the photon energy of 532 nm incident photon. As a result, the peaks located at around 1420 and 3115 cm^−1^ in the Raman spectra correspond to the ZPL of NV^0^ and NV^−^ centers in the PL spectra. Differently from the Raman scattering, the fluorescence emission intensity of NV^0^ and NV^−^ centers depend not only on the power of the excitation photons but also the concentration of NV centers in diamond. The intensity ratio of NV^0^ centers to the first-order Raman phonon line of diamond (diamond peak) is decreased from 0.41 to 0.02 when the input N_2_ gas flow increases from 0.05 to 10 sccm, and that of the NV^−^ centers to the diamond peak decreases from 1.39 to 0.13, respectively, as shown in Figure 1e–h. The decrease in the intensity ratio indicates the decrease in NV center concentration in diamond, which can be attributed to the formation of other types of nitrogen in diamond, such as pairs or clusters, instead of NV centers, leading to the decrease in NV center concentrations in diamond.

### 3.2. Raman and PL Characterization of NV Centers under Different Temperatures

**Figure 2:**
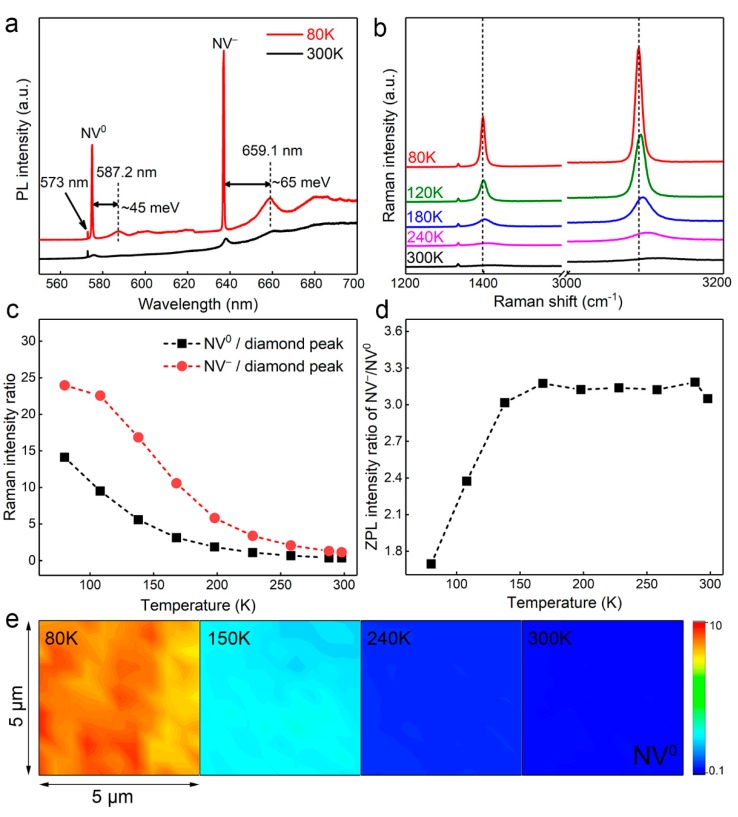
(**a**) PL spectra and (**b**) Raman spectra of the diamond sample with temperature measured from 80 K to 300 K. The zero-phonon line (ZPL) intensity ratio of nitrogen-vacancy (NV) centers to (**c**) the diamond first-order Raman phonon line, (**d**) the ZPL intensity ratio of NV^−^ to NV^0^ from 80 K to 300 K, and (**e**) Raman mapping of the ratio of the ZPL intensity of the NV^0^ center to diamond peak with a size of about 5 μm × 5 μm.

The PL spectra of the diamond sample (Sample B) with NV^0^ and NV^−^ centers were measured at 80 K and 300 K, as shown in Figure 2. According to the PL spectra shown in Figure 2a, the ZPL intensity of NV centers is very weak at 300 K, which can be attributed to the suppressing of the fluorescence emission of NV centers by temperature-caused strong phonon scattering. When the temperature decreases to 80 K, the intensity of diamond peak stays nearly constant, whereas the ZPL intensity of the NV centers becomes very strong. Additionally, two broad bands at 587.2 and 659.1 nm can also be observed which correspond to a-phonon-related photon sidebands of NV^0^ and NV^−^ centers with an energy shift of 45 and 65 meV, respectively [37,38]. The increase in the ZPL intensity and the appearance of phonon sidebands of NV centers indicate the suppression of phonon scattering and the strengthening of the fluorescence emission of NV centers in diamond at low temperature.

The Raman spectra of diamond samples (Sample B) under different temperatures are shown in Figure 2b. When the temperature increases from 80 K to 300 K, the intensity of the diamond peak in the Raman spectra is nearly constant, indicating the weak temperature influence on Raman scattering. However, the ZPL intensity of NV centers increases dramatically with a decrease in temperature from 300 K to 80 K. Meanwhile, the ZPL linewidth of NV centers get sharper and also accompanies the left shift of NV center positions. The ratio of the ZPL intensity of NV^0^ and NV^−^ centers to the diamond peak between 80 K and 300 K is shown in Figure 2c, and can be seen to decrease significantly with the increase in temperature. Moreover, the ratio of the NV^−^ centers to the diamond peak decreases faster than that relating to the NV^0^ centers. It can be also observed in Figure 2d that the ZPL intensity ratio of NV^−^ to NV^0^ shows a linear relationship to temperature when the temperature is smaller than 180 K, and tends to be constant in the high temperature region. This may be attributed to the temperature-induced electron-acoustic phonon interaction effect, which induces a stronger suppression of the fluorescence emission NV^0^ centers than of the NV^−^ centers in the higher temperature region. The Raman mapping of the ZPL intensity ratio of the NV^0^ centers to the diamond peak under each temperature are shown in Figure 2e. The fluorescence intensity ratios in the 5 μm × 5 μm area are all in the range of 7.64–9.64, 2.142–2.92, 0.502–0.698, and 0.245–0.404 when the testing temperature is 80, 150, 240, and 300 K, respectively. As the fluorescence intensity of NV centers only depends on the concentrations of NV centers when the power of excitation laser is constant, the color of the Raman mapping in each temperature indicates the near uniform distribution of NV centers in the CVD diamond sample.

### 3.3. The Dependence of Energy Level Shifts of NV Centers to Temperature

**Figure 3:**
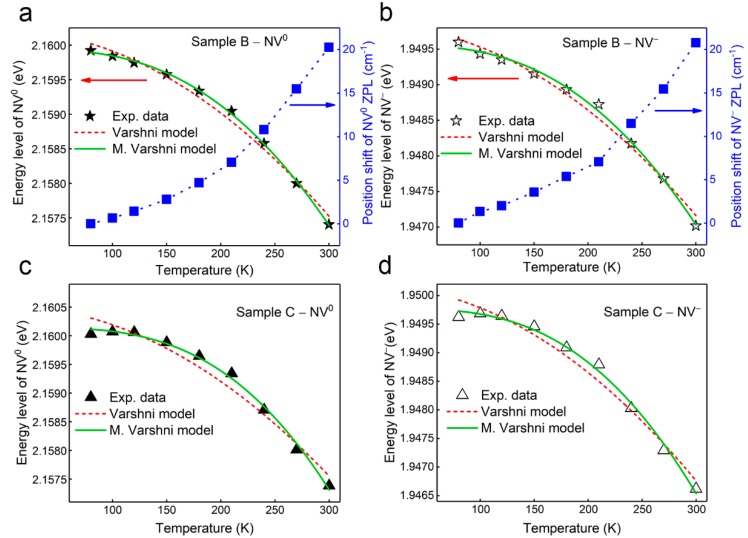
The ZPL position and energy level variation of (**a**) NV^0^ and (**b**) NV^−^ centers with temperature for diamond sample Sample B; the energy level variation of (**c**) NV^0^ and (**d**) NV^−^ centers with temperature for diamond sample Sample C.

The Raman position and energy levels of NV^0^ and NV^−^ centers at different temperatures were calculated and have been replotted in Figure 3 based on Figure 2b. As shown in Figure 3a,b, when the temperature increases from 80 K to 300 K, the positions of the ZPL of the NV^0^ and NV^−^ centers shift positively by about 21 cm^−1^, which corresponds to a decrease in energy level of about 2.6 meV. For conventional semiconductors, like Si, Ge, and GaAs, the dependence of the energy gap shifts on temperature follows the Varshni empirical model [33], i.e.,

(4) $$E_{\left(T\right)}=E_{0}\;-\;\frac{\alpha T^{2}}{T+\beta }$$

where *α* is the constant, *β* is related to the Debye temperature (*θ_D_*), and *E*_0_ is the energy gap at 0 K. The Varshni equation was proposed by Y.P. Varshni to describe a relation for the variation in the energy gap (Eg) with temperature (T) in semiconductors, and it is believed that most of the variation in the energy gap arises from the following two mechanisms [33]: (1) a shift in the relative position of the conduction and valence bands due to the temperature-dependent dilatation of the lattice, and (2) a shift in the relative position of the conduction and valence bands due to a temperature-dependent electron lattice interaction, which forms the major contribution to the variation.

However, the Varshni model cannot successfully describe the temperature dependence of NV centers’ energy level shifts in diamond, and the fitting curve overlap well with the experimental data, as shown in Figure 3a,b. The reason why the Varshni model does not work well is due to the unique properties of diamond, such as its high Debye temperature (*θ_D_*: 2200 K) [33] and large optical phonon frequency (165.2 meV) [34]. Moreover, because the thermal expansion coefficient of diamond (1.6 × 10^−6^ K^−1^) is much smaller than that of conventional semiconductors, like Si (3.6 × 10^−6^ K^−1^) and Ge (6 × 10^−6^ K^−1^), it has been demonstrated that the electron-acoustic interaction is the major mechanism for the energy level shifts of NV centers in diamond instead of the thermal expansion effect [39]. As a result, the modified Varshni (M. Varshni) formula proposed by Li et. al. [39] has been used to describe the temperature dependence of the ZPL of the NV^0^ and NV^−^ centers in diamond, i.e.,

(5) $$E_{\left(T\right)}=E_{0}\;-\;\frac{AT^{4}}{{\left(T+B\right)}^{2}}$$

where *A* and *B* are constants. The temperature dependence of energy level shifts of NV centers can be fitted by both the Varshni and modified Varshni models, as shown in Figure 3a,b and Table 1. The fitting line obtained by the modified Varshni model presents a better overlap with the experimental data than that of the Varshni model, with *R*^2^ = 0.999 and 0.996 for the NV^0^ and NV^−^ centers, respectively. Moreover, when the temperature tends to 0 K, the ZPLs of the NV^0^ and NV^−^ centers are about 2.15685 eV [28] and 1.94688 eV [39], as mentioned in previous reports, which are much closer to the values obtained using the modified Varshni model. In addition, the modified Varshini model is also applicable for NV centers in diamond sample Sample C, as shown in Figure 3c,d, indicating that the relationship of the energy level shifts of NV centers to temperature is independent of nitrogen concentration in CVD diamond.

### 3.4. The Determination of NV Centers in Temperature Measurement

**Figure 4:**
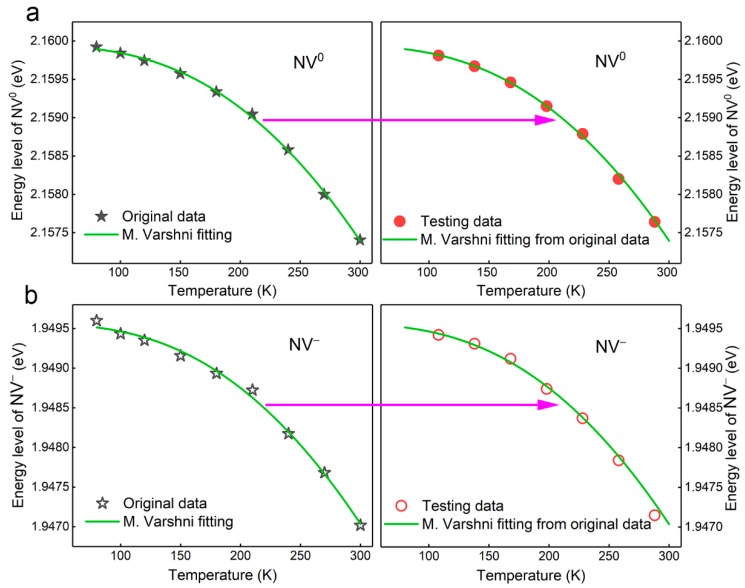
The accuracy determination of (**a**) NV^0^ and (**b**) NV^−^ centers in temperature measurement based on the energy level shifts.

According to the temperature dependence of the energy level shifts of NV centers, diamond can be used for temperature measurement based on the fluorescence emission of NV centers, as shown in Figure 4. As discussed above, the relationship of the energy level of NV centers to temperature can be calculated using the modified Varshni model based on the measured data (original data), as shown in Figure 4a,b. Another set of data (testing data), which was measured at a different temperature, locate well relative to the modified Varshni fitting curves obtained from the original data. The calculated working temperatures for the diamond sample were determined based on the specific modified Varshni fitting curves from the original data, as shown in Table 2. The calculated temperature is in good agreement with the temperature measured by thermocouple, revealing about ±2% and ±3% errors for the NV^0^ and NV^−^ centers, respectively.

## 4. Conclusions

In summary, the temperature-dependent behavior of NV centers was investigated in this work using Raman and PL spectroscopy. Both the intensity and the linewidth of the zero-phonon line of NV centers showed high dependence on environmental temperature. The energy level shifts of NV centers in diamond can be better explained using a modified Varshni model than the traditional one, and the electron-acoustic interaction is the major mechanism for the energy level shifts in diamond. According to the dependence of energy level shifts on temperature, a diamond temperature sensor with high accuracy has been proposed. It was found that diamond shows high precision in temperature detection with only ±2% and ±3% error for NV^0^ and NV^−^ centers, respectively. Compared to other diamond thermometers based on the ODMR of NV^−^ centers, this work does not have a high requirement of experimental facilities. The investigation of this work helps us comprehensively understand the influence of temperature on energy level shifts in diamond, and it also shows the potential of NV centers in temperature detection and monitoring in biosystems.

## Figures and Tables

**Table 1:** A comparison of the relationship between energy levels of NV^0^ and NV^−^ centers fitted by the Varshni and modified Varshni models.

**Varshni Model**		***E*_0_ (eV)**	***α* (eV/K)**	***β* (K)**	***R*^2^**
$\;E\left(T\right)=E_{0}\;-\;\frac{\alpha T^{2}}{T+\beta }$	NV^0^	2.16021	1.68396 × 10^5^	5.64457 × 10^12^	0.98848
	NV^−^	1.94983	1.79467 × 10^5^	6.06312 × 10^12^	0.98676
**Modified Varshni Model**		***E*_0_ (eV) **	***A* (eV/K^2^) **	***B* (K)**	***R*^2^**
$E\left(T\right)=E_{0}\;-\;\frac{AT^{4}}{{\left(T+B\right)}^{2}}$	NV^0^	2.15994	6.94965 × 10^−8^	170.30905	0.99918
	NV^−^	1.94956	6.72722 × 10^−8^	164.62925	0.99605

**Table 2:** A comparison of the accuracy between setting and calculated temperature of NV centers based on the modified Varshni model.

	Setting temperature (K)	108	138	168	198	228	258	288
NV^0^	Calculated temperature (K)	110.1	138.8	167.6	198.2	225.7	261.3	289.1
	Error	1.94%	0.58%	−0.24%	0.10%	−1.01%	1.28%	0.38%
NV^−^	Calculated temperature (K)	109.8	135.6	162.7	200.9	228.7	260.8	294.9
	Error	−1.67%	−1.74%	−3.15%	1.46%	0.31%	1.08%	2.40%

* Fitting parameters of modified Varshni equation for NV^0^ and NV^−^ centers: NV^0^: *E*_0_: 2.15994 (eV); A: 6.94965 × 10^−8^ (eV/K^2^); B: 170.30905 (K); NV^−^: *E*_0_: 1.94956 (eV); A: 6.72722 × 10^−8^ (eV/K^2^); B: 164.62925 (K).

## Supplementary Materials

The following are available online at https://www.mdpi.com/2079-4991/9/11/1576/s1, Figure S1: UV-visible spectra of three diamond samples (A, B, C); Table S1: The calculated absorption coefficient and the nitrogen concentrations of three diamond samples.

## References

[1] Wrachtrup J., Jelezko F. (2006). Processing quantum information in diamond. J. Phys. Condens. Matter.

[2] Liu G.Q., Pan X.Y. (2018). Quantum information processing with nitrogen-vacancy centers in diamond. Chin. Phys. B.

[3] Gruber A., Drabenstedt A., Tietz C., Fleury L., Wrachtrup J., von Borczyskowski C. (1997). Scanning confocal optical microscopy and magnetic resonance on single defect centers. Science.

[4] Fuchs G.D., Dobrovitski V.V., Toyli D.M., Heremans F.J., Awschalom D.D. (2009). Gigahertz Dynamics of a Strongly Driven Single Quantum Spin. Science.

[5] Jelezko F., Gaebel T., Popa I., Domhan M., Gruber A., Wrachtrup J. (2004). Observation of coherent oscillation of a single nuclear spin and realization of a two-qubit conditional quantum gate. Phys. Rev. Lett..

[6] Jelezko F., Wrachtrup J. (2004). Read-out of single spins by optical spectroscopy. J. Phys. Condens. Matter.

[7] Gurudev Dutt M.V., Childress L., Jiang L., Togan E., Maze J., Jelezko F., Zibrov A.S., Hemmer P.R., Lukin M.D. (2007). Quantum register based on individual electronic and nuclear spin qubits in diamond. Science.

[8] Maze J.R., Stanwix P.L., Hodges J.S., Hong S., Taylor J.M., Cappellaro P., Jiang L., Dutt M.V.G., Togan E., Zibrov A.S. (2008). Nanoscale magnetic sensing with an individual electronic spin in diamond. Nature.

[9] Acosta V.M., Bauch E., Ledbetter M.P., Waxman A., Bouchard L.S., Budker D. (2010). Temperature dependence of the nitrogen-vacancy magnetic resonance in diamond. Phys. Rev. Lett..

[10] Acosta V.M., Bauch E., Ledbetter M.P., Santori C., Fu K.M.C., Barclay P.E., Beausoleil R.G., Linget H., Roch J.F., Treussart F. (2009). Diamonds with a high density of nitrogen-vacancy centers for magnetometry applications. Phys. Rev. B.

[11] Tetienne J.P., Hingant T., Kim J.V., Diez L.H., Adam J.P., Garcia K., Roch J.F., Rohart S., Thiaville A., Ravelosona D. (2014). Nanoscale imaging and control of domain-wall hopping with a nitrogen-vacancy center microscope. Science.

[12] Beams R., Smith D., Johnson T.W., Oh S.H., Novotny L., Vamivakas A.N. (2013). Nanoscale Fluorescence Lifetime Imaging of an Optical Antenna with a Single Diamond NV Center. Nano Lett..

[13] Pfaff W., Hensen B.J., Bernien H., van Dam S.B., Blok M.S., Taminiau T.H., Tiggelman M.J., Schouten R.N., Markham M., Twitchen D.J. (2014). Unconditional quantum teleportation between distant solid-state quantum bits. Science.

[14] Doherty M.W., Struzhkin V.V., Simpson D.A., McGuinness L.P., Meng Y., Stacey A., Karle T.J., Hemley R.J., Manson N.B., Hollenberg L.C. (2014). Electronic properties and metrology applications of the diamond NV^−^ center under pressure. Phys. Rev. Lett..

[15] Sotoma S., Epperla C.P., Chang H.C. (2018). Diamond Nanothermometry. Chemnanomat.

[16] Davies G., Hamer M.F. (1976). Optical Studies of the 1.945 eV Vibronic Band in Diamond. Proc. R. Soc. A Math. Phys. Eng. Sci..

[17] Mita Y. (1996). Change of absorption spectra in type-Ib diamond with heavy neutron irradiation. Phys. Rev. B.

[18] Loubser J., Vanwyk J.A. (1978). Electron-Spin Resonance in Study of Diamond. Rep. Prog. Phys..

[19] Davies G. (1979). Dynamic Jahn-Teller distortions at trigonal optical-centers in diamond. J. Phys. C Solid State Phys..

[20] Breeding C.M., Shigley J.E. (2009). The “TYPE” Classification System of Diamonds and its Importance in Gemology. Gems Gemol..

[21] Meijer J., Burchard B., Domhan M., Wittmann C., Gaebel T., Popa I., Jelezko F., Wrachtrup J. (2005). Generation of single-color centers by focused nitrogen implantation. Appl. Phys. Lett..

[22] Dyer H.B., Preez L.D. (1965). Irradiation Damage in Type I Diamond. J. Chem. Phys..

[23] Ermakova A., Pramanik G., Cai J.M., Algara-Siller G., Kaiser U., Weil T., Tzeng Y.K., Chang H.C., McGuinness L.P., Plenio M.B. (2013). Detection of a Few Metallo-Protein Molecules Using Color Centers in Nanodiamonds. Nano Lett..

[24] Hsiao W.W.W., Hui Y.Y., Tsai P.C., Chang H.C. (2016). Fluorescent Nanodiamond: A Versatile Tool for Long-Term Cell Tracking, Super-Resolution Imaging, and Nanoscale Temperature Sensing. Acc. Chem. Res..

[25] Kask P., Palo K., Ullmann D., Gall K. (1999). Fluorescence-intensity distribution analysis and its application in biomolecular detection technology. Proc. Natl. Acad. Sci. USA.

[26] Doherty M.W., Acosta V.M., Jarmola A., Barson M.S.J., Manson N.B., Budker D., Hollenberg L.C.L. (2014). Temperature shifts of the resonances of the NV^−^ center in diamond. Phys. Rev. B.

[27] Plakhotnik T., Doherty M.W., Cole J.H., Chapman R., Manson N.B. (2014). All-Optical Thermometry and Thermal Properties of the Optically Detected Spin Resonances of the NV^−^ Center in Nanodiamond. Nano Lett..

[28] Chen X.D., Dong C.H., Sun F.W., Zou C.L., Cui J.M., Han Z.F., Guo G.C. (2011). Temperature dependent energy level shifts of nitrogen-vacancy centers in diamond. Appl. Phys. Lett..

[29] Bhosale J., Ramdas A.K., Burger A., Munoz A., Romero A.H., Cardona M., Lauck R., Kremer R.K. (2012). Temperature dependence of band gaps in semiconductors: Electron-phonon interaction. Phys. Rev. B.

[30] Odonnell K.P., Chen X. (1991). Temperature dependence of semiconductor band gaps. Appl. Phys. Lett..

[31] Kingsmith R.D., Needs R.J., Heine V., Hodgson M.J. (1989). A First-Principle Calculation of the Temperature Dependence of the Indirect Band-Gap of Silicon. EPL (Europhys. Lett.).

[32] Lautenschlager P., Allen P.B., Cardona M. (1985). Temperature Dependence of Band Gaps in Si and Ge. Phys. Rev. B.

[33] Varshni Y.P. (1967). Temperature dependence of energy gap in semiconductors. Physica.

[34] Klein C.A., Hartnett T.M., Robinson C.J. (1992). Critical-point phonon frequencies of diamond. Phys. Rev. B.

[35] Sato T., Ohashi K., Sudoh T., Haruna K., Maeta H. (2002). Thermal expansion of a high purity synthetic diamond single crystal at low temperatures. Phys. Rev. B.

[36] Nath N.N. (1935). The dynamical theory of the diamond lattice. I. Proc. Indian Acad. Sci. Sect. A.

[37] Wang K.Y., Li Z.H., Zhang B., Zhu Y.M. (2012). Investigation of vibronic structures of optical centres in diamond by photoluminescence spectra. Acta Phys. Sin..

[38] Wang K., Steeds J.W., Li Z., Tian Y. (2016). Photoluminescence Studies of Both the Neutral and Negatively Charged Nitrogen-Vacancy Center in Diamond. Microsc. Microanal..

[39] Li C.C., Gong M., Chen X.D., Li S., Zhao B.W., Dong Y., Guo G.C., Sun F.W. (2017). Temperature dependent energy gap shifts of single color center in diamond based on modified Varshni equation. Diam. Relat. Mater..

