# Supplementary material for: A Diamond Temperature Sensor Based on the Energy Level Shift of Nitrogen-Vacancy Color Centers
Source: Nanomaterials (Basel). 2019 Nov 7;9(11):1576. doi: 10.3390/nano9111576 (PMC6915693; doi:10.3390/nano9111576)
Supplement: Supplementary file 1 [file nanomaterials-09-01576-s001.pdf]

Supplementary Materials

Article

A Diamond Temperature Sensor Based on the Energy Level Shift of Nitrogen-Vacancy Color Centers

Mingyang Yang [1,2,†], Qilong Yuan [1,2,*,†], Jingyao Gao [1,2], Shengcheng Shu [1,2], Feiyue Chen [1,3], Huifang Sun [1,4], Kazuhito Nishimura [5], Shaolong Wang [1], Jian Yi [1], Cheng-Te Lin [1,2,*] and Nan Jiang [1,2,*]

1 Key Laboratory of Marine Materials and Related Technologies, Zhejiang Key Laboratory of Marine Materials and Protective Technologies, Ningbo Institute of Materials Technology and Engineering (NIMTE), Chinese Academy of Sciences, Ningbo 315201, China; yangmingyang@nimte.ac.cn (M.Y.); gaojingyao@nimte.ac.cn (J.G.); shushengcheng@nimte.ac.cn (S.S.); chenfeiyue@nimte.ac.cn (F.C.); sunhuifang@nimte.ac.cn (H.S.); wangshaolong@nimte.ac.cn (S.W.); yijian@nimte.ac.cn (J.Y.)
2 Center of Materials Science and Optoelectronics Engineering, University of Chinese Academy of Sciences, Beijing 100049, China
3 College of Science, Henan University of Technology, Zhengzhou 10463, China
4 Faculty of Materials Science and Engineering, Kunming University of Science and Technology, Kunming 650093, China
5 Advanced Nano-processing Engineering Lab, Mechanical Engineering, Kogakuin University, Tokyo 192-0015, Japan; nishimura@cc.kogakuin.ac.jp
* Correspondence: yuanqilong@nimte.ac.cn (Q.Y.); linzhengde@nimte.ac.cn (C.-T.L.); jiangnan@nimte.ac.cn (N.J.)
† These authors contributed equally to this work.

1.

The concentration of nitrogen impurities in diamond can be estimated based on the absorption at 270 nm on the UV-Visible spectra [1], as shown in Figure S1. And the nitrogen concentration (N_S) (ppm) is proportional to the absorption coefficient at 270 nm (α_S) (cm^{-1}) [2]:

$$N_S = 0.56\alpha_S$$

According to the UV-Visible spectra, the calculated absorption coefficient and the nitrogen concentrations of three diamond samples is shown in Table S1. The diamond samples have been labeled using the concentration of nitrogen atoms in Figure 1. And a brief introduction of the calculation of nitrogen concentration in diamond has been also added as a supporting information.

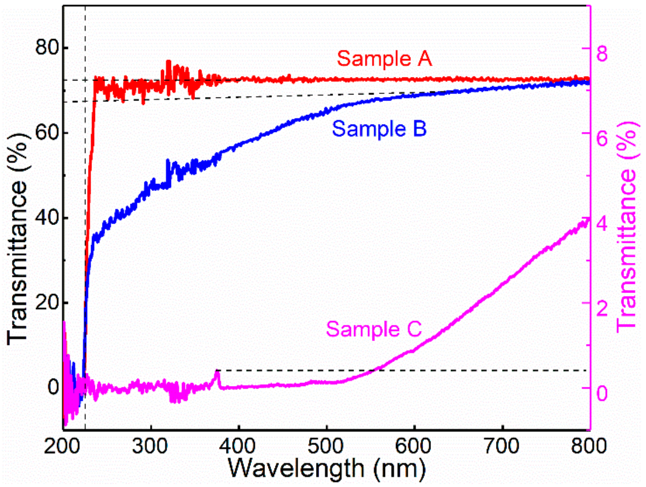

Figure S1: UV-Visible spectra of three diamond samples (A, B, C)

Table S1: The calculated absorption coefficient and the nitrogen concentrations of three diamond samples

Samples	A	B	C
Transmittance at 270 nm	0.711	0.404	0.001
Diamond thickness (mm)	0.552	0.191	0.286
Absorption coefficient: α_S (cm^{-1})	6.2	47.7	241.5
Nitrogen concentration: N_S (ppm)	3.5	26.7	127.7

Reference

1. Chrenko, R.M.; Strong, H.M.; Tuft, R.E. Dispersed paramagnetic nitrogen content of large laboratory diamonds. *Philosophical Magazine* **1971**, *23*, 313–318.
2. Sumiya, H.; Satoh, S. High-pressure synthesis of high-purity diamond crystal. *Diamond and Related Materials* **1996**, *5*, 1359–1365.
